# Supplementary material for: Autologous Antibody Responses to an HIV Envelope Glycan Hole Are Not Easily Broadened in Rabbits
Source: J Virol. 2020 Mar 17;94(7):e01861-19. doi: 10.1128/JVI.01861-19 (PMC7081899; doi:10.1128/JVI.01861-19)
Supplement: Supplemental file 1 [file JVI.01861-19-s0001.pdf]

Supplementary Information

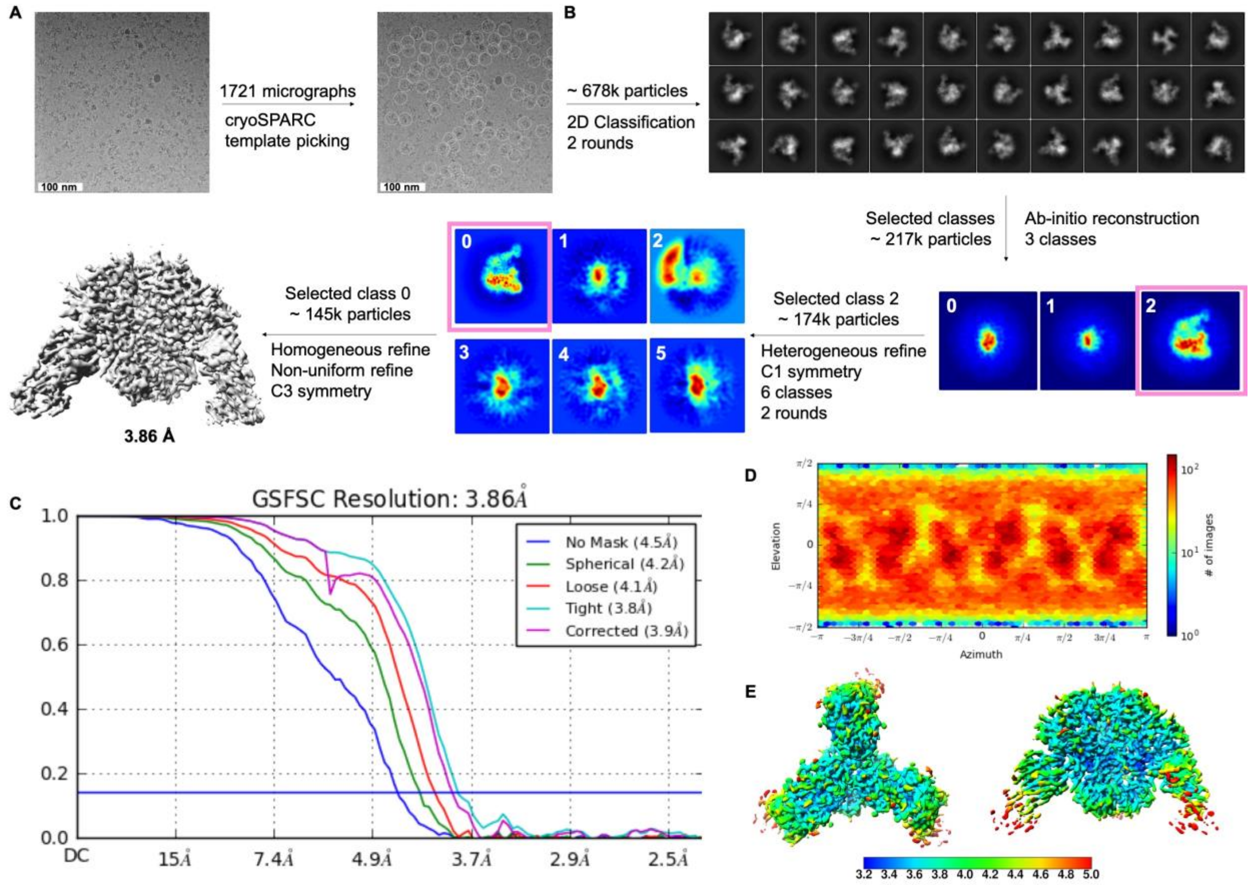

FIG S1. CryoEM data processing for the B41-13B complex. (A) Representative aligned and dose-weighted cryo-EM micrograph of B41-13B complex in vitreous ice, circles showing particles picked up by cryosparc v2. template picking. (B) Cryo-EM data processing scheme to obtain final reconstruction. (C) FSC plots of unmasked (blue) and masked (pink) reconstructions. (D) Relative angular distribution of final reconstruction used for model building. Red bars represent views with more particles. (E) Cryo-EM map colored according to local resolution determined by local resolution function in cryoSPARC v2. Color key shows local resolution in Å.

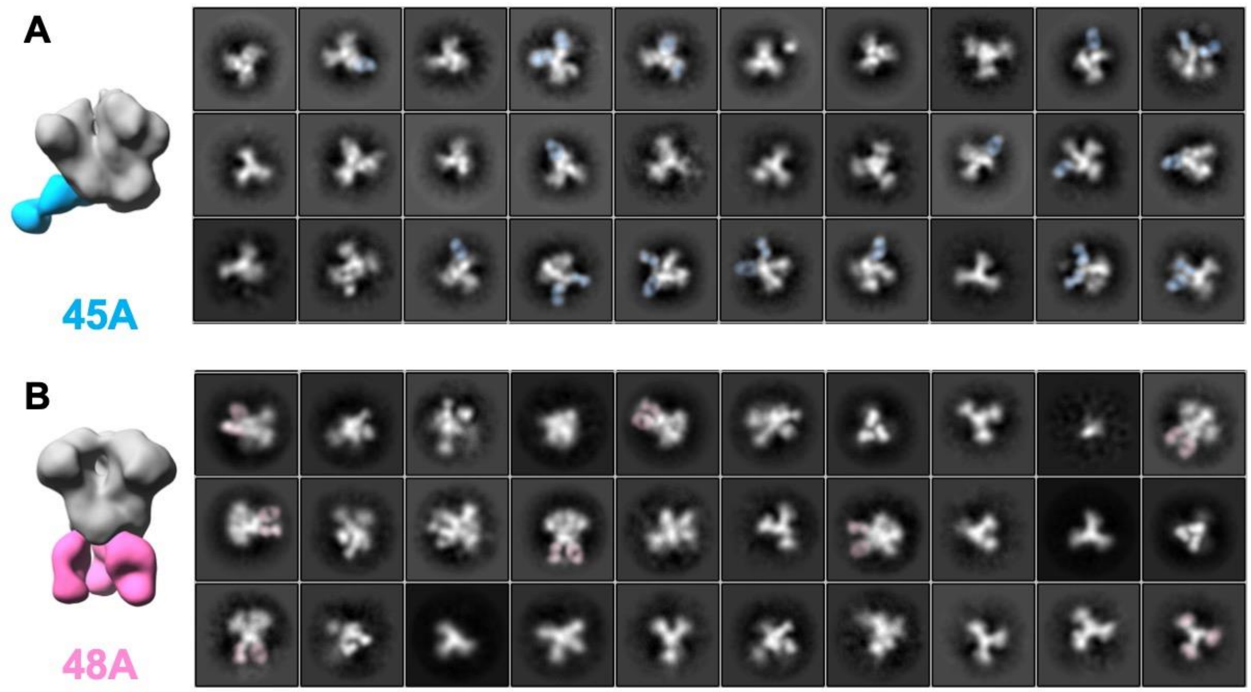

FIG S2. Negative stain EM 2D classification using cryoSPARC. Trimers are shown in complex with base binding antibodies 45A (blue) or 48A (pink). Classes that resemble complexes were selected for 3D reconstruction (left panel), C1 symmetry is applied for B41-45A complex and C3 is applied for B41-48A complex.

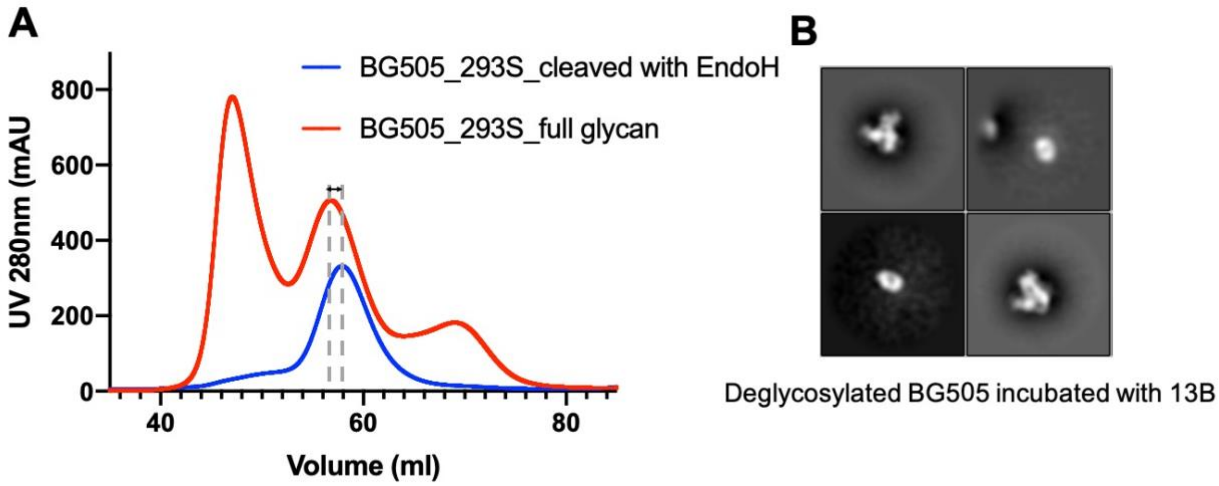

FIG S3. Deglycosylated BG505 characterization. (A) SEC characterization of deglycosylated BG505. HEK 293S expressed BG505 was purified over a 2G12 column and then by SEC (red line). The SEC-purified sample was then treated with EndoH overnight and then purified through the same column (blue). (B) Representative NS-EM 2D classes of 10-fold excess 13B antibody incubated with deglycosylated BG505 overnight at RT.

pseudovirus	mAb						
	13A	13B	13B2	16A	16B	16C	16D
QH0692.42	>50	>50	>50	>50	>50	>50	>50
1006_11_C3_1601	>50	>50	>50	>50	>50	>50	>50
1054_07_TC4_1499	>50	>50	>50	>50	>50	>50	>50
1012_11_TC21_3257	>50	>50	>50	>50	>50	>50	>50
ZM214M.PL15	>50	>50	>50	>50	>50	>50	>50
246F C1G	>50	>50	>50	>50	>50	>50	>50
Q461.e2	>50	>50	>50	>50	>50	>50	>50
T257-31	>50	>50	>50	>50	>50	>50	>50
263-8	>50	>50	>50	>50	>50	>50	>50
T250-4	>50	>50	>50	>50	>50	>50	>50
T278-50	>50	>50	>50	>50	>50	>50	>50
X1193_C1	>50	>50	>50	>50	>50	>50	>50
X2088_C9	>50	>50	>50	>50	>50	>50	>50
6041.v3.c23	>50	>50	>50	>50	>50	>50	>50
6540.v4.c1	>50	>50	>50	>50	>50	>50	>50
6545.v4.c1	>50	>50	>50	>50	>50	>50	>50

FIG S4. Neutralization analysis of selected viruses lacking glycan sites at 289 from the 117-virus panel against B41-specific mAbs.

	16A-biotin				16B-biotin				16C-biotin				16D-biotin			
16A	32	30	29	82	93	88	90	95	64	58	43	112	14	13	10	81
16B	3	3	3	85	9	11	11	96	3	3	3	88	5	5	5	104
16C	9	9	30	95	58	57	91	99	7	8	22	90	6	6	7	87
16D	54	44	61	79	94	92	96	96	65	63	78	92	14	13	20	81
13A	4	3	5	92	15	11	30	100	4	5	5	109	5	5	6	107
13B	11	12	15	99	46	65	77	101	21	20	24	88	7	7	8	102
49A	5	4	12	97	18	23	64	99	6	7	12	89	6	5	7	99
46A	4	4	74	106	18	23	100	100	6	6	76	124	5	5	14	111
blank	101	101	101	96	97	103	102	98	98	100	100	101	107	105	82	106
45A	98	101	105	104	98	103	101	97	89	99	99	93	107	115	100	127
48A	101	97	90	95	97	103	100	95	96	100	99	92	94	106	102	109
Hybrid	100	98	101	96	97	101	99	96	90	90	91	91	97	97	96	93

	13A-biotin				13B-biotin				46A-biotin				49A-biotin			
16A	93	95	93	98	63	60	60	91	77	75	67	98	91	90	85	100
16B	14	11	18	106	5	5	10	89	7	7	9	97	9	9	12	109
16C	50	49	87	102	16	17	49	88	28	28	60	98	40	40	80	103
16D	95	91	95	94	79	66	88	87	89	86	92	98	95	95	97	100
13A	11	9	26	120	4	4	9	85	6	6	7	97	7	7	9	111
13B	60	57	77	110	15	16	25	84	26	28	34	97	37	37	46	102
49A	17	15	56	120	5	5	18	83	8	8	16	97	8	8	16	106
46A	22	20	99	120	6	6	86	87	8	7	79	98	11	10	92	110
blank	99	98	102	101	103	104	105	89	100	101	100	99	99	100	101	100
45A	99	99	100	101	92	89	93	86	101	99	98	98	99	100	100	100
48A	99	100	100	97	104	94	103	89	102	101	102	98	99	101	99	100
Hybrid	95	96	96	97	93	94	94	93	103	101	100	99	99	101	100	100

	PG9-biotin				PGT121-biotin				8ANC195-biotin				PGV04-biotin			
16A	175	182	179	176	112	115	114	110	88	85	80	95	100	98	95	99
16B	161	171	167	179	109	111	110	110	80	74	79	96	98	99	97	100
16C	166	178	178	174	110	112	111	108	78	78	80	97	101	102	100	102
16D	178	182	174	160	117	118	115	111	99	93	95	99	107	107	106	104
13A	107	122	145	146	93	107	112	111	73	70	81	94	93	99	101	103
13B	104	115	125	135	83	93	109	109	23	23	30	90	48	57	65	98
49A	103	114	127	139	76	102	109	109	10	11	15	90	68	74	86	100
46A	161	178	177	171	113	117	116	111	78	75	85	97	105	108	101	101
blank	102	112	80	105	99	98	101	103	100	98	107	95	101	100	101	98
45A	120	134	112	144	101	99	104	106	81	83	92	99	103	101	100	99
48A	58	72	75	114	79	81	96	101	75	79	96	97	92	90	98	97
SELF	22	30	43	90	11	15	28	58	9	46	94	106	9	36	69	91

FIG S5. Competition ELISAs of B41-spsecific mAbs and human bnAbs PG9, PGT121, 8ANC195, and PGV04. Competition is expressed as percentage binding where 100% was the absorbance measured when B41 SOSIP protein only was captured on the anti-avi-tag ELISA plate. The non-HIV specific mAb hybrid was used as a negative control for non-specific inhibition of biotinylated rabbit mAb binding. The unbiotinylated version of each human bnAb (PG9, PGV04, PGT121, 8ANC195) was used as a positive control (referred to as SELF) only in the assay where the binding of each of these bnAbs in biotinylated form was being assessed.

Map	B41 SOSIP + 13B Fab
**Data collection**	
Microscope	FEI Talos Arctica
Voltage (kV)	200
Detector	Gatan K2 Summit
Recording mode	Counting
Nominal magnification	36,000
Movie micrograph pixelsize (Å)	1.15
Dose rate (e^-/[(camera pixel)*s])	5.67
Number of frames per movie micrograph	56
Frame exposure time (ms)	250
Movie micrograph exposure time (s)	14
Total dose (e^-/Å^2)	60
Defocus range (μm)	-2.0 to -0.5
**EM data processing**	
Number of movie micrographs	1,721
Number of molecular projection images in map	147,520
Symmetry	C3
Map resolution (FSC 0.143; Å)	3.9
Local resolution range (Å)[1]	3.2 – 5.3
Map sharpening B-factor (Å^2)	-198
**Structure Building and Validation**	
Number of atoms in deposited model	
B41 gp120	10686
B41 gp41	2925
Fab Fv	5220
Glycans	1611
MolProbity score	1.18
Clashscore	2.67
Map correlation coefficient	0.81
EMRinger score	2.60
RMSD from ideal	
Bond length (Å)	0.019 (35)
Bond angles (˚)	1.77 (72)
Ramachandran plot	
Favored (%)	97.33
Allowed (%)	2.54
Outliers (%)	0.13
Side chain rotamer outliers (%)	0.72

[1]In modeled regions of map; based on Cryosparc 2 Local Resolution function

Table S1. Cryo-EM data collection and refinement statistics.
